# Correction: Schwartz et al. Cleaning Strategies of Synthesized Bioactive Coatings by PEO on Ti-6Al-4V Alloys of Organic Contaminations. *Materials* 2023, *16*, 4624

**DOI:** 10.3390/ma18091989

**Published:** 2025-04-28

**Authors:** Avital Schwartz, Alexey Kossenko, Michael Zinigrad, Viktor Danchuk, Alexander Sobolev

**Affiliations:** 1 Department of Chemical Engineering, Ariel University, Ariel 4070000, Israel; avitals@ariel.ac.il (A.S.); kossenkoa@ariel.ac.il (A.K.); zinigrad@ariel.ac.il (M.Z.); 2 Physics Department, Faculty of Natural Sciences, Ariel University, Ariel 4076414, Israel; viktorde@ariel.ac.il

In the original publication [1], there were duplications in Figure 6 as published. To eliminate this error, a repeated procedure for analyzing cross-sections of the same samples was performed. The corrected Figure 6 appears below. The authors state that the scientific conclusions are unaffected. This correction was approved by the Academic Editor. The original publication has also been updated.

## Figures and Tables

**Figure 6:**
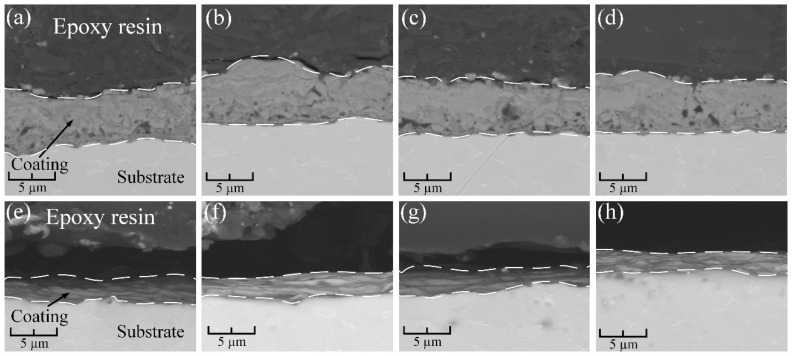
Cross-section morphology for samples after surface cleaning: (**a**) AS-A; (**b**) AS-A-UV; (**c**) AS-A-P; (**d**) AS-A-O; (**e**) MS-A; (**f**) MS-A-UV; (**g**) MS-A-P; (**h**) MS-A-O.
